# Excellent Dark/Light Dual-Mode Photoresponsive Activities Based on g-C_3_N_4_/CMCh/PVA Nanocomposite Hydrogel Using Electron Beam Radiation Method

**DOI:** 10.3390/molecules28227544

**Published:** 2023-11-11

**Authors:** Jin-Yu Yang, Dong-Xu Tang, Dong-Liang Liu, Kun Liu, Xiao-Jie Yang, Yue-Sheng Li, Yi Liu

**Affiliations:** 1 Hubei Key Laboratory of Radiation Chemistry and Functional Materials, School of Nuclear Technology and Chemistry & Biology, Hubei University of Science and Technology, Xianning 437100, China; 18727791310@163.com (J.-Y.Y.); tdtd800@126.com (D.-X.T.); ldl142325@163.com (D.-L.L.); 17754119632@163.com (K.L.); mailyangxiaojie@126.com (X.-J.Y.); 2 Key Laboratory of Coal Conversion and New Carbon Materials of Hubei Province, School of Chemistry and Chemical Engineering, Wuhan University of Science and Technology, Wuhan 430081, China; 3 College of Chemistry and Chemical Engineering, Tiangong University, Tianjin 300387, China; yiliuchem@whu.edu.cn

**Keywords:** g-CP hydrogel, electron beam radiation method, photocatalytic degradation, photocatalytic antibacterial, cell activity

## Abstract

Photocatalytic technology for inactivating bacteria in water has received much attention. In this study, we reported a dark–light dual-mode sterilized g-C_3_N_4_/chitosan/poly (vinyl alcohol) hydrogel (g-CP) prepared through freeze–thaw cycling and an in situ electron-beam radiation method. The structures and morphologies of g-CP were confirmed using Fourier infrared spectroscopy (FTIR), X-ray diffraction spectroscopy (XRD), X-ray photoelectron spectroscopy (XPS), scanning electron microscopy (SEM), solid ultraviolet diffuse reflectance spectroscopy (UV-vis DRS), and Brunauer–Emmett–Teller (BET). Photocatalytic degradation experiments demonstrated that 1 wt% g-CP degraded rhodamine B (RhB) up to 65.92% in 60 min. At the same time, g-CP had good antimicrobial abilities for *Escherichia coli* (*E. coli*) and *Staphylococcus aureus* (*S. aureus*) within 4 h. The shapes of g-CP were adjustable (such as bar, cylinder, and cube) and had good mechanical properties and biocompatibility. The tensile and compressive modulus of 2 wt% g-CP were 0.093 MPa and 1.61 MPa, respectively. The Cell Counting Kit-8 (CCK-8) test and Hoechst33342/PI double staining were used to prove that g-CP had good biocompatibility. It is expected to be applied to environmental sewage treatment and wound dressing in the future.

## 1. Introduction

The economy is growing fast, but there are still some thorny problems. Firstly, the discharge of dyes’ wastewater can cause serious pollution to the environment. After light exposure, dyes will consume a large amount of oxygen in water, resulting in hypoxia and affecting the growth of aquatic organisms and microorganisms [1,2]. Secondly, overuse of antibiotics can lead to bacterial resistance. The high rates of isolating clinically resistant strains and the complex mechanisms of resistance contribute to the challenges associated with treating bacterial infections [3,4,5]. Because conventional methods do not solve the above problems well, photocatalytic technology is considered an effective method for degrading pollutants and inactivating bacteria [6,7,8]. Photocatalytic technology has the advantages of simple operation, rapid reaction, and no secondary pollution, which promotes its applications in dye degradation and bacterial inactivation [9,10].

Hydrogel consisting of a three-dimensional network structure has been applied in tissue engineering [11], drug slow release [12], antibacterial [13], and other fields. The preparation methods of hydrogel include physical methods, chemical methods, and radiation methods. The physical method mainly uses hydrophobic association, hydrogen bonding, or electrostatic interaction to cross-link it, so its mechanical properties are poor [14]. Although the hydrogel prepared using chemical methods solves the above shortcomings, it needs the addition of a crosslinking agent and initiator. The toxicity of initiators and crosslinking agents can limit the application of hydrogel in biomedical fields [15]. Electron beam radiation is a green synthesis method, which mainly uses free radicals generated after water is radiated to initiate the crosslinking of polymers [16]. In addition, the hydrogel prepared through radiation has high mechanical properties and controllable shapes.

Graphite-phase carbon nitride (g-C_3_N_4_) is an excellent photocatalytic material with simple preparation, cheap raw materials, non-toxicity, and good catalytic activity. It has a broad application prospect in energy storage, hydrogen production, nitrogen fixation, and pollutants removal [17,18,19]. Nano-sized g-C_3_N_4_ is not conducive to recovery and reuse. The typical carriers are activated carbon, ceramic, and zeolite, among which hydrogel stands out. Hydrogel has the advantages of porosity, high permeability, and easy swelling, making it an excellent biomimetic photocatalytic reactor [20,21,22]. For example, Hao et al. prepared g-C_3_N_4_ hydrogel microspheres exhibiting good photocatalytic performance by chelating calcium ions and sodium alginate [23]. Thurston et al. used sulfuric acid, glutaraldehyde, and polyvinyl alcohol (PVA) to form g-C_3_N_4_-based hydrogel exhibiting photocatalytic antibacterial properties [24].

Chitosan is widely distributed in nature and is an excellent natural polymer [25]. Carboxymethyl chitosan (CMCh) is one of its common derivatives. It is a good antibacterial agent [26]. At present, many studies have focused on the development of carboxymethyl-chitosan-based antibacterial hydrogels. Wahid [27] et al. prepared CMCS/copper oxide hydrogel, which had good swelling and antibacterial properties. Kang [28] et al. prepared vinyl carboxymethyl chitosan thermo-sensitive hydrogel for drug release.

In this paper, g-C_3_N_4_/CMCh/PVA (g-CP) hydrogel was prepared through freezing–thawing and electron beam radiation, which solved the disadvantage of the poor antibacterial ability of g-C_3_N_4_ in the dark. The structures and properties of g-CP were investigated using infrared spectroscopy (FTIR), scanning electron microscopy (SEM), and solid ultraviolet visible diffuse reflection (UV-vis DRS). The photocatalytic degradation of RhB by 1 wt% g-CP was 65.92% in 60 min. g-CP had good antimicrobial properties against *Escherichia coli* (*E. coli*) and *Staphylococcus aureus* (*S. aureus*). In addition, this hydrogel had good biocompatibility. Most importantly, g-CP had good mechanical properties and shape controllabilities (such as strip, cylinder, and cube), thus expanding its application range in environmental and biomedical fields.

## 2. Results and Discussion

### 2.1. FTIR Analysis

**Figure 1:**
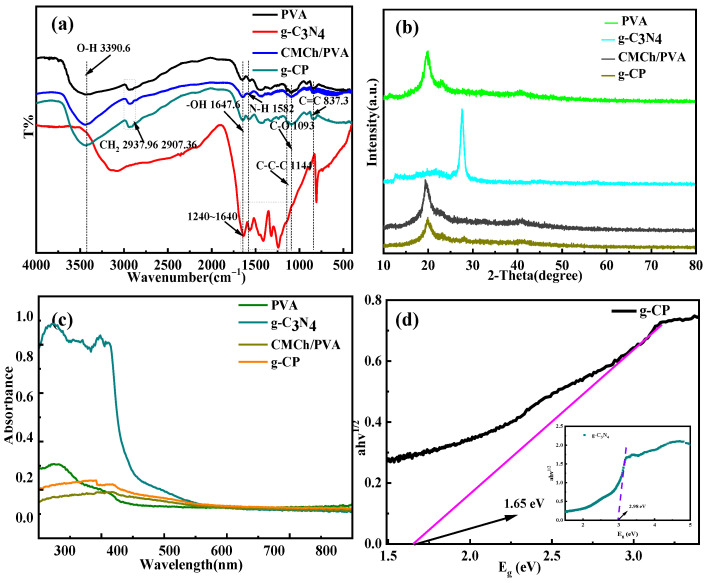
(**a**–**c**) FTIR, XRD, and UV-Vis spectra of the four materials, respectively; (**d**) the band gap diagram of g-C_3_N_4_ and 2 wt% g-CP.

The FTIR spectra of Figure 1a showed the presence of functional groups of the prepared materials. In the spectra of PVA, the absorption peaks of 3390.6 cm^−1^, 2937.96 cm^−1^, 2907.36 cm^−1^, 1420.04 cm^−1^, and 1086.63 cm^−1^ were respectively attributed to the -OH (ν_OH-PVA_), -CH_2_ (ν_asCH2 PVA_), -CH_2_ (ν_sCH2 PVA_), -CH_2_ (δ_CH2 PVA_), and -C-O (ν_C-O PVA_). The absorption peak at 1647.6 cm^−1^ corresponded to the superposition of the C=C stretching vibration and the hydroxyl group deformation vibration peaks in RHC=CH_2_. The absorption peak at 1144 cm^−1^ was contributed by the C-C-C stretching vibration of PVA. The absorption peak at 837.3 cm^−1^ corresponded to the C=C stretching vibration at the end of the vinyl [29]. The absorption peaks at 1582 cm^−1^, 1409 cm^−1^, 1310 cm^−1^, and 1053 cm^−1^ corresponded to the symmetric stretching vibration and asymmetric stretching vibration of the carboxymethyl of CMCh, and the stretching vibration of C-N and C-O bonds, respectively [30]. In the spectrum of g-C_3_N_4_, the absorption peak at 810 cm^−1^ corresponded to the out-of-plane bending mode of heterocyclic C-N, and the stretching vibrational peaks of aromatic C-N were at 1200–1600 cm^−1^ [31]. The spectrum of g-CP contained the characteristic functional groups of chitosan and carbon nitride.

### 2.2. XRD Analysis

Figure 1b showed the XRD patterns to test the crystal phase structure of all samples. For PVA, the wide peak at about 20° corresponded to its semi-crystalline characteristics [32]. The diffraction peak at 13.1° and 27.4° corresponded to the (100) and (002) crystal plane of g-C_3_N_4_. Diffraction peaks of g-C_3_N_4_ in composite hydrogel were covered by PVA. The reason for this phenomenon might be the low content of g-C_3_N_4_ in the composite hydrogel or the weak crystallinity [33].

### 2.3. UV-Vis Analysis

Figure 1c,d showed the UV-Vis spectra and band gap diagrams of the samples, respectively. The optical absorption wavelength of g-C_3_N_4_ was about 414.7 nm and the band gap width was 2.98 eV. The optical absorption wavelengths of PVA and PVA/CMCh were larger compared to g-C_3_N_4_. Therefore, when g-C_3_N_4_ was loaded in PVA/CMCh hydrogel, its absorption wavelength was redshifted. At the same time, the band gap width of 2 wt% g-CP narrowed to 1.65 eV, which promoted its absorption in visible light.

### 2.4. XPS Analysis

**Figure 2:**
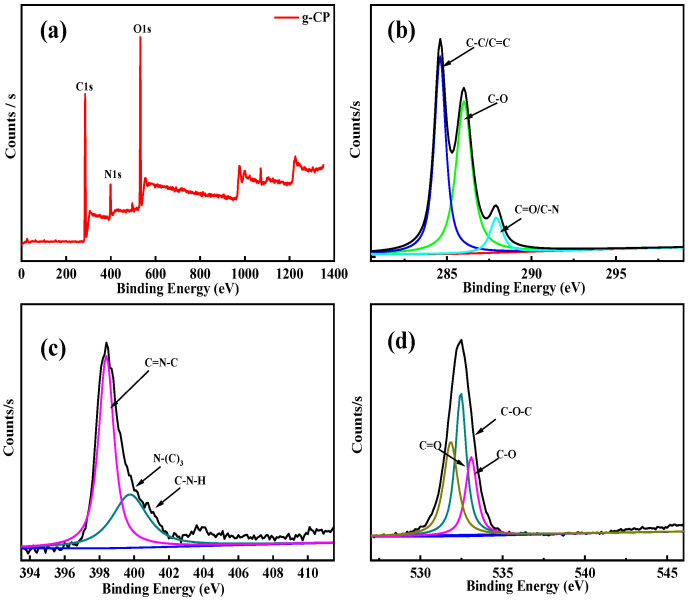
(**a**–**d**) XPS full spectrum, and C1s, N1s, and O1s spectra of 2wt% g-CP, respectively.

Figure 2a–d showed the full spectrum of 2 wt% g-CP and the single energy spectra of C1s, N1s, and O1s, respectively. The C1s, N1s, and O1s peaks of g-C_3_N_4_ appeared at about 285, 400, and 520 eV, respectively. The binding energy at 284.6 eV corresponded to the C-C bond of the g-C and the C=C peak of the alkene at the end of the PVA [34]. The binding energy at 287.9 eV belonged to the C-O and C-N bonds of 2 wt% g-CP. The binding energies at 398.7 eV, 399.8 eV, and 400.8 eV corresponded to the C=N-C, H-N-(C)_3_, and C-N-H bonds of the g-C_3_N_4_ triazine ring, respectively [35]. The binding energy peaks at 531.8, 532.5, and 533 eV were characteristic peaks of CMCh, corresponding to C=O, C-O-C, and C-O bonds, respectively [36].

### 2.5. SEM Analysis

**Figure 3:**
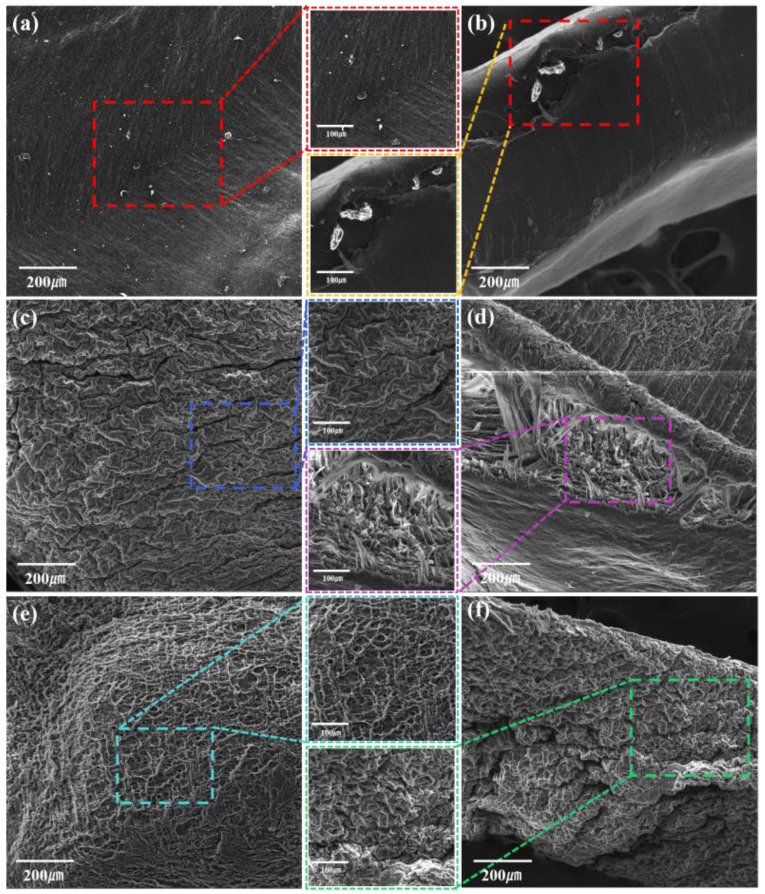
The front and side SEM images of PVA (**a**,**b**), CMCh/PVA (**c**,**d**), and 2wt% g-CP (**e**,**f**) hydrogels.

The front and cross-sections of PVA, CMCh/PVA, and 2 wt% g-CP hydrogels were characterized using scanning electron microscopy. As could be seen from Figure 3a–f, the cross-sectional images of the g-CP showed more continuous porous structures compared to the monolithic and binary hydrogels. It was proved that the addition of g-C_3_N_4_ made the hydrogel have a more compact network structure and a higher degree of cross-linking. There might be three main reasons for the denser network of g-CP: firstly, the addition of g-C_3_N_4_ could increase the quantity of crosslinking points, thus forming a greater number of crosslinked networks; secondly, g-C_3_N_4_ had high hardness and strength, which could be used as a reinforcing agent to strengthen the skeleton structure of the hydrogel; thirdly, powder could effectively fill the pores within the hydrogel, and this physical blocking effect would result in a more compact crosslinked network, reducing the porosity of the material. These would potentially shorten the molecular transport channel and greatly enhance the molecular adsorption kinetics of RhB.

### 2.6. TEM Analysis

**Figure 4:**
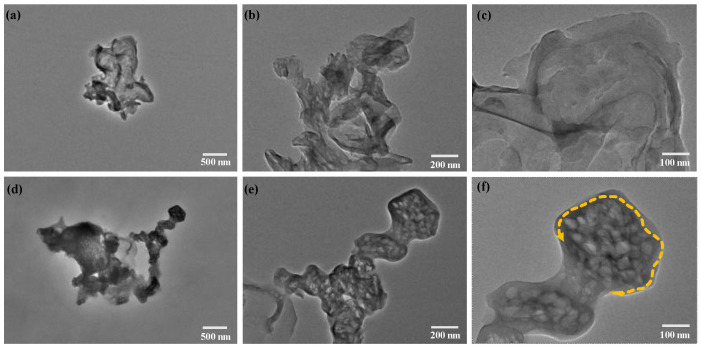
TEM images of g-C_3_N_4_ (**a**–**c**) and g-CP (**d**–**f**).

g-C_3_N_4_ and g-CP were characterized using transmission electron microscopy. As shown in Figure 4a–c, g-C_3_N_4_ presented a nanoparticle layer structure with holes. When nanomaterials were synthesized into the hydrogel, the dispersion was poor. g-CP showed the stacking phenomenon, as shown in Figure 4 d,e. In addition, it could be seen from Figure 4f that g-CP had significantly more holes than g-C_3_N_4_. Moreover, there was a single layer of amorphous form around g-CP, which might be the polymers.

### 2.7. BET Analysis

**Figure 5:**
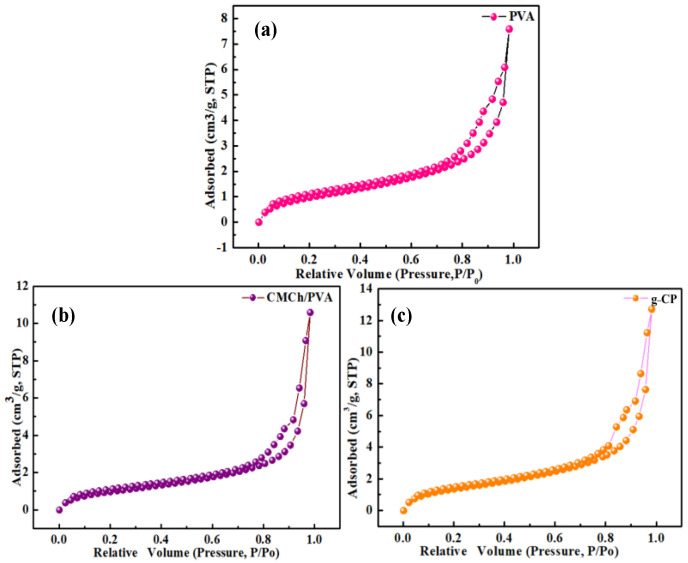
Nitrogen adsorption–desorption isotherms of PVA (**a**), CMCh/PVA (**b**), 2 wt% g-CP (**c**) hydrogels.

To examine the variation in pore characteristics of the samples, N_2_ adsorption–desorption isotherms were obtained. Compared with unitary and binary hydrogels, 2 wt% g-CP had a larger specific surface area and pore volume and smaller pore size (Figure 5a–c and Table 1). The measured results were consistent with those of SEM.

### 2.8. Mechanical Performance Analysis

**Figure 6:**
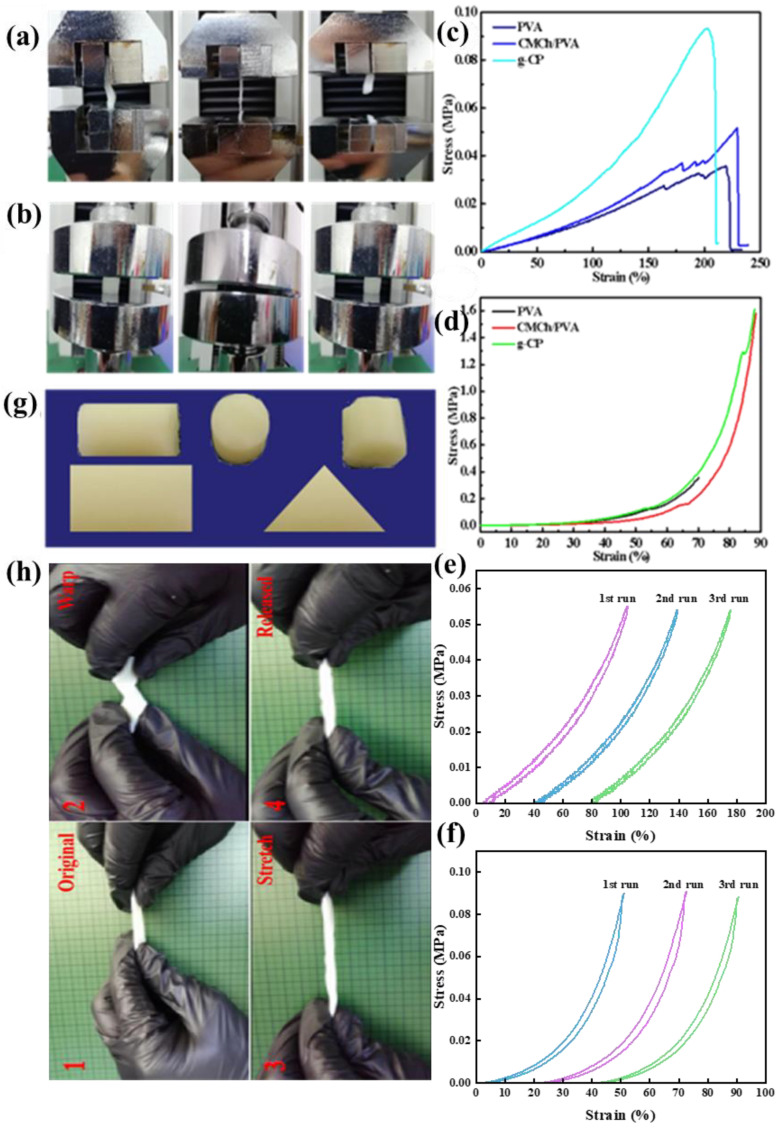
Test diagram of engineering performance. The stress–strain test diagrams of 2 wt% g-CP (**a**–**f**); the pictures of different geometric shapes and flexibility test drawings, respectively (**g**,**h**).

Mechanical properties are one of the basic properties of engineering hydrogels. The mechanical properties of PVA, CMCh/PVA, and 2 wt% g-CP hydrogels were tested using an electronic universal testing machine (QJ-210, Shanghai Qingji Instrument Technology, Shanghai, China). Figure 6a–d showed that the tensile and compressive moduli of PVA, CMCh/PVA, and 2 wt% g-CP hydrogels were 0.033 MPa, 0.0424 MPa, and 0.093 MPa and 0.357 MPa, 1.58 MPa, and 1.61 MPa, respectively. g-CP had better tensile and compressive moduli than the mono and binary hydrogels. Figure 6e,f showed the cyclic stretching and cyclic compression experiments. The hysteresis curves tended to be stable through tensile and cyclic experiments, which indicated that g-CP had good resilience.

g-CP could be prepared into different geometries and applied in various aspects, as shown in Figure 6g. The flexibilities of g-CP were tested using torsion and tensile experiments. As shown in Figure 6h, there was no fracture when the rotation and elongation of g-CP exceeded 200%. After removing the external force, the g-CP could be completely restored to its original length. Notably, g-CP showed excellent rebound abilities when external forces were removed.

### 2.9. Adsorption–Photocatalytic Degradation of RhB

Adsorption experiments were performed in order to determine the equilibrium time for the dark adsorption of RhB on PVA, CMCh/PVA, and g-CP hydrogels.

The adsorption capacity q_e_ (mg g^−1^) can be calculated as follows:

q_e_ = V(C_0_ − C_e_)/m (1)

where C_0_ (mg L^−1^) is the initial concentration of RhB. C_e_ (mg L^−1^) represents the equilibrium concentration after adsorption. V (mL) and m (g) represent the volume of RhB and mass of the hydrogel, respectively. The pseudo-first-order kinetic model and pseudo-second-order kinetic model of Lagergren [37] were used to analyze the adsorption rate data. The kinetic equations are as follows:

ln(q_e_ − q_t_) = ln q_e_ − k_1_t (2)

t/q_t_ = 1/(k_2_q_e_^2^) + t/q_e_ (3)

where q_e_ (mg g^−1^) and q_t_ (mg g^−1^) represent the adsorption capacity of the hydrogel at equilibrium and different times, respectively. k_1_ (min^−1^) and k_2_ (g mg^−1^ min^−1^) are pseudo-first-order and pseudo-second-order adsorption rate constants, respectively.

**Figure 7:**
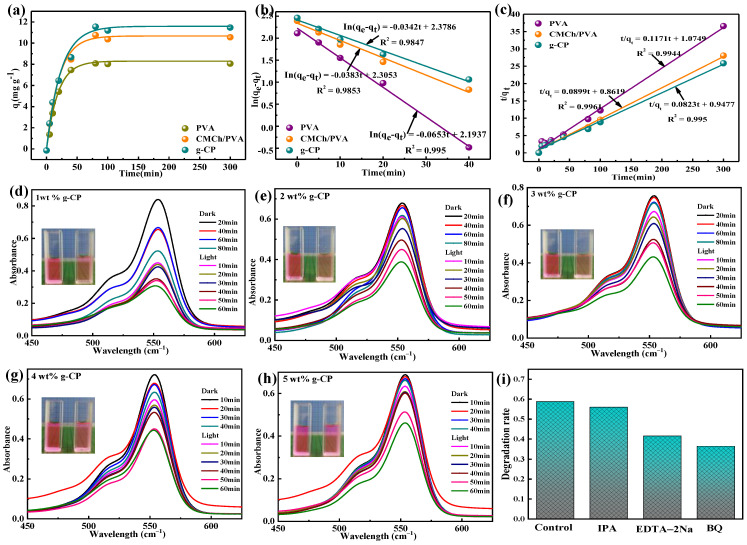
Performance tests of adsorption and photocatalysis. The fitting curves, the first-order kinetic fitting and second-order kinetic fitting curves of the adsorption capacity, and the time of RhB by three hydrogels, respectively (**a**–**c**); the UV absorption spectra of RhB adsorption–photocatalytic degradation by hydrogel with different g-C_3_N_4_ contents (**d**–**h**); free radical trapping diagram of g-CP (**i**).

As shown in Figure 7a, the maximum adsorption capacities of PVA, CMCh/PVA, and g-CP hydrogels were 8.23 mg g^−1^, 10.9 mg g^−1^, and 11.69 mg g^−1^, respectively (C_0_ = 50 mg L^−1^, V = 50 mL, m = 0.5 g, T = 298 K). All the hydrogels reached adsorption equilibrium within 80 min. g-CP had the highest adsorption capacity probably related to the increase in specific surface area. According to the dynamic fitting curves and correlation coefficients (Table 2), the R_2_ of the quasi-second-order dynamic model was larger than that of the quasi-first-order dynamic model (Figure 7 b,c). The results of quasi-second-order fitting were more consistent with the experiments. Therefore, the adsorption process of g-CP on RhB showed chemisorption [38]. Of course, this might also be caused by the physical adsorption expansion of hydrogel [39]. As shown in Figure 7d–h, the degradation rates of RhB by 1–5 wt% g-CP were 65.92%, 56.83%, 51.18%, 49.78%, and 47.74%, respectively. Among them, 1 wt% g-CP showed the highest degradation rate. The possible reason was that when the masses of g-C_3_N_4_ were the same, the content of 1 wt% g-CP was the largest. Therefore, the existence of hydrogel increased the adsorption capacity and improved the catalytic efficiency. In order to study the involvement of reactive species in the photocatalytic reaction process, p-benzoquinone (BQ), ethylenediaminetetraacetic acid disodium salt (EDTA-2Na), and isopropanol (IPA) were, respectively, introduced as the scavengers of superoxide radicals (•O_2_^−^), holes (h+), and hydroxyl radicals (•OH) to examine the effects of reactive species on the photocatalytic degradation of RhB. From Figure 7i, the BQ and EDTA-2Na led to an obvious suppression of the degradation efficiency of RhB. However, the IPA had little impact on the degradation efficiency. The results indicated that •O_2_^−^ and h^+^ played a major role in the degradation of RhB.

### 2.10. Zone of Inhibition and Photocatalytic Antibacterial Experiments

**Figure 8:**
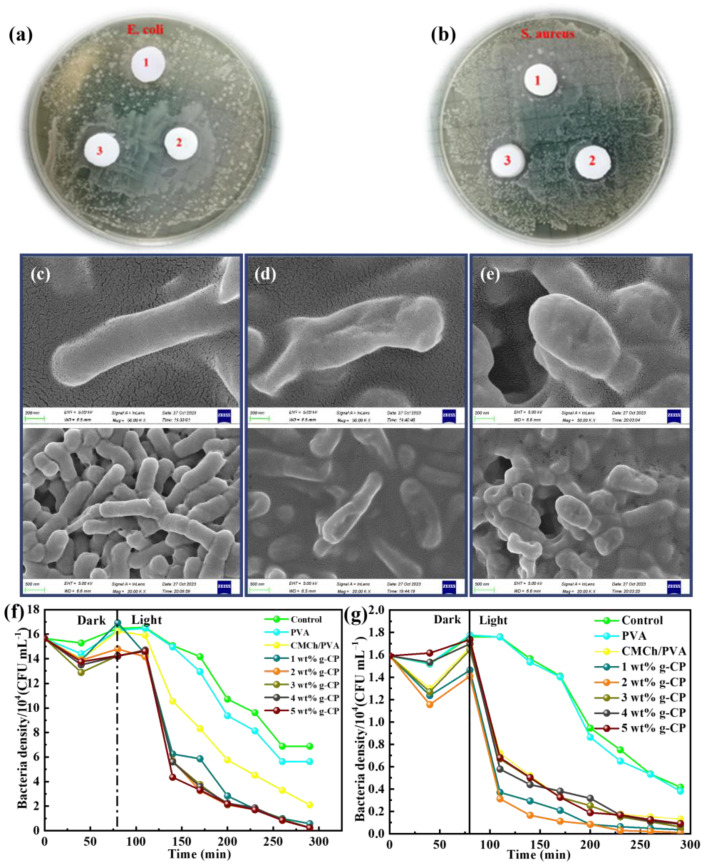
Zone of inhibition and photocatalytic antibacterial tests. Inhibition of *E. coli* and *S. aureus* by different hydrogels under dark conditions in pictures (1: PVA, 2: CMCh/PVA, 3: g-CP), respectively (**a**,**b**); SEM images of g-CP against *E. coli* at 1 h, 2 h, and 12 h, respectively (**c**–**e**); the PVA, CMCh/PVA, and different amounts of g-CP hydrogels on the photocatalytic antibacterial performance of *E. coli* and *S. aureus* (**f**,**g**).

The antibacterial properties of the materials were confirmed using zone of inhibition and photosensitive antibacterial experiments. It could be seen from Figure 8a,b that the antibacterial effect of g-CP was obviously better than those of mono and binary hydrogels. Taking *E. coli* as an example, the physical antibacterial properties of g-CP against it were recorded using SEM. Figure 8c–e showed the bacterial morphology of g-CP at 1 h, 2 h, and 12 h, respectively. As can be seen from the figures, the original *E. coli* had a good rod-like structure and a smooth appearance. Over time, *E. coli* appeared to shrink and even break. These were consistent with the results of the bacteriostatic zone experiment. The addition of CMCh and g-C_3_N_4_ significantly improved the weak antibacterial performance of PVA. The -NH_3_^+^ and -NHCH_2_COOH on the CMCh had an electrostatic interaction and chelating ability with bacteria, which could destroy the stability of the outer membrane to inactivate it [40].

Photocatalytic antimicrobial experiments showed that the higher g-C_3_N_4_ content of the hydrogel gradually enhanced the inhibition effect on *E. coli* (Figure 8f,g). The reason for this result might be that the cell wall structures of the two bacteria were different. *S. aureus* is a typical Gram-positive bacterium with a thick cell wall, which is mainly composed of peptidoglycan. On the contrary, Gram-negative bacteria have only a thin layer of peptidoglycan in the cell wall [41]. The amount of ROS produced by g-CP increased with the increase in g-C_3_N_4_. ROS could penetrate the bacterial wall of E. coli to directly or indirectly disrupt its respiratory and physiological activities. The 2 wt% g-CP showed the best antibacterial effect against *S. aureus*. *S. aureus* had a thick cell wall and was insensitive to ROS produced by g-C_3_N_4_ [42]. CMCh exerted a significant antibacterial effect against *S. aureus*. The interaction of NH_3_^+^ of CMCh with anions in the microbial cell wall hindered the reproduction. Therefore, 2 wt% g-CP demonstrated a synergistic antibacterial effect against *S. aureus*. In addition, the bacterial concentration in surface water generally did not exceed 10^3^ CFU mL^−1^ [43], which was much lower than 10^5^ CFU mL^−1^. Therefore, g-CP would be more efficient in sterilizing bacteria in practical applications. Compared with nanoparticles, g-CP was easier to recycle and showed more potential in practical applications.

### 2.11. Cytotoxicity Analysis

**Figure 9:**
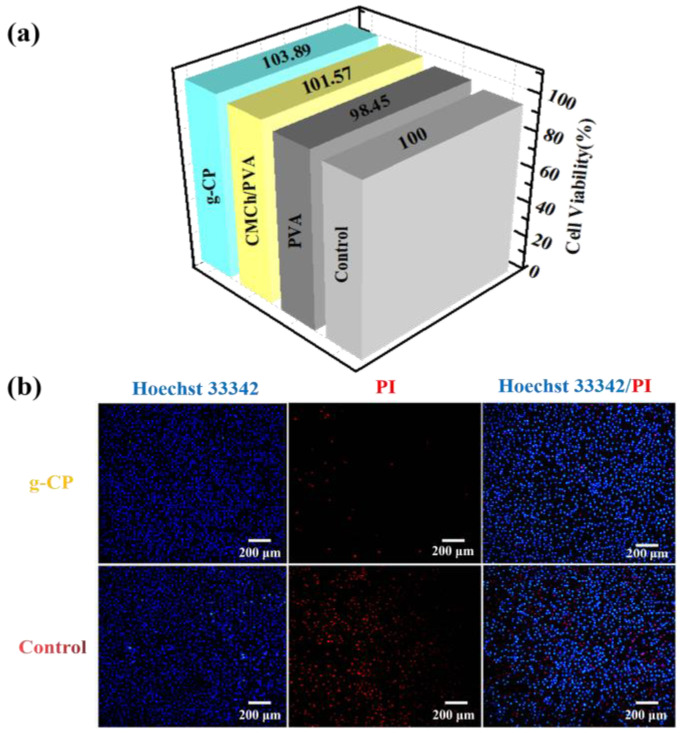
Cytotoxicity test of 2 wt% g-CP. The MTT assay map of 2 wt% g-CP on L929 cells (**a**); the toxicity images of 2 wt% g-CP by Hoechst33342/PI cell staining (**b**).

Cytotoxicity was also one of the key factors in evaluating engineered materials. The effect of hydrogels on L-929 cell viability was assessed using CCK-8 and Hoechst 33342/PI double staining (Figure 9a,b). The CCK-8 assay demonstrated that PVA, CMCh/PVA, and 2 wt% g-CP hydrogels had a small effect on the viability of L929 cells, which were 100%, 98.45%, and 101.57%, respectively. The 2 wt% g-CP promoted the proliferation of L929 cells with a cell viability of 103.89%. In addition, the Hoechst 33342/PI double staining (blue color after Hoechst 33342 staining and red color after PI staining) assay also confirmed that the cell density of 2 wt% g-CP was increased relative to the control group. Toxicology experiments both fully confirmed that g-CP had good biocompatibility. Meanwhile, the toxicological experiments also indirectly confirmed that electron beam radiation was a green preparation method.

### 2.12. Photocatalytic Degradation and Antibacterial Mechanism of g-CP

The mechanism of photocatalytic degradation of g-CP was the decomposition of RhB into carbon dioxide and water by active species such as superoxide radicals (O_2_^−^•) and holes (h^+^) generated by g-C_3_N_4_. The good antibacterial properties of g-CP were mainly classified into the following two aspects. Firstly, the physical antibacterial effect: the positive charge (amino group) in the CMCh molecule interacted with the negative charge on the surface of bacteria, which led to the leakage of bacterial proteins and other components, thus achieving the antibacterial effect [44]. Secondly, the photocatalytic antibacterial effect: Under sunlight, g-C_3_N_4_ in g-CP would produce free radicals, which would further react with O_2_ to form ROS. ROS reacted with phospholipids of the bacterial membrane, which led to membrane destruction and interfered with the normal metabolism of cells, thus achieving the bactericidal effect [45].

## 3. Experimental Section

### 3.1. Materials

Carboxymethyl chitosan (CMCh), polyvinyl alcohol (PVA), urea, and nitric acid (HNO_3_) were from Sinopharm Chemical Reagent Co., Ltd., Shanghai, China; peptone, yeast extract powder, beef extract, and agar powder were provided by Sigma Company; *E. coli* (CCTCCAB91112) and *S. aureus* (CCTCCAB910393) were from Wuhan University.

### 3.2. Preparation of Samples

#### 3.2.1. Preparation of g-C_3_N_4_

An amount of 10 g of urea was heated at 550 °C for 180 min with a heating rate of 5 °C min^−1^ to obtain yellow powder. Then, g-C_3_N_4_ was washed three times with 0.1 mol L^−1^ HNO_3_ and purified water, and dried at 60 °C for 24 h.

#### 3.2.2. Radiation Construction of g-CP

**Scheme 1:**
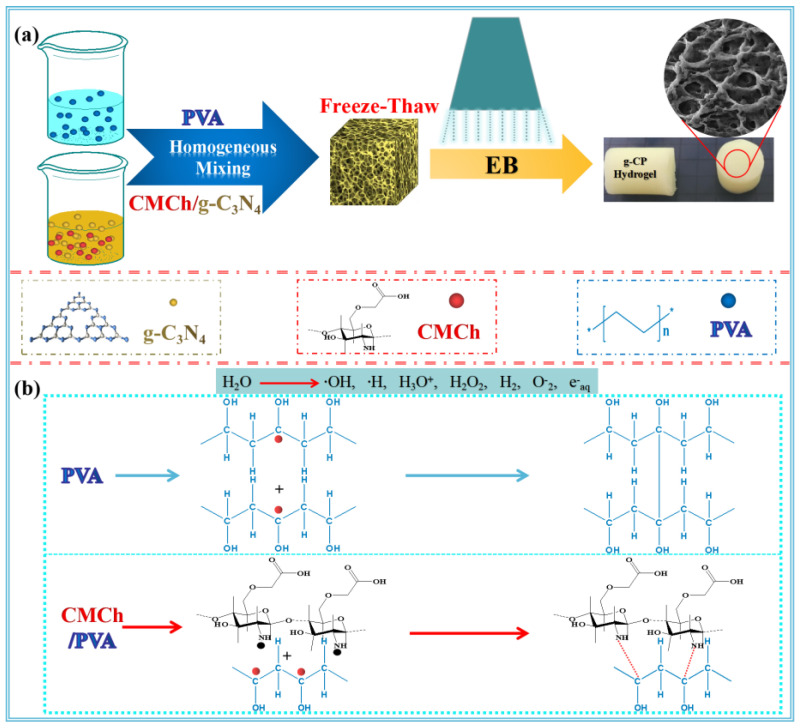
Diagram of preparation process (**a**) and radiation synthesis mechanism of g-CP (**b**).

The 10% aqueous solution of PVA was heated to 85 °C and stirred for 2 h. Different masses of g-C_3_N_4_ were added into 10 mL water for ultrasonic dispersion. Then, 0.5 g of CMCh was gradually added to the above solutions, stirred, and dissolved to obtain g-C_3_N_4_/CMCh solution. The above solution was thoroughly mixed, frozen, and thawed three times. The samples were radiated with a 1 MeV electron accelerator (Wasik Associates, Boston, MA, USA). The absorbed radiation dose was 30 kGy, and the dose rate was 5 kGy/pass. Finally, the samples with different mass fractions (1–5 wt%) were obtained by soaking in deionized water three times and freeze-drying (Scheme 1a). The preparation methods of single hydrogel (PVA) and binary hydrogel (PVA/CMCh) were similar to the above method. The mechanism of polymer crosslinking might be divided into the following two steps. Firstly, water molecules were irradiated by an electron beam to produce free radicals. Finally, the free radical excited PVA to self-polymerize and cross-link with CMCS to form a network structure (Scheme 1b).

### 3.3. Material Characterization

The structures of the materials were qualitatively analyzed using Fourier transform infrared spectroscopy (FTIR, Thermo Fisher Nicolet, Waltham, MA, USA). The crystal structure was characterized using X-ray diffraction (XRD, Shimadzu, Rigaku, Japan). The morphology was studied using scanning electron microscopy (SEM, TFiS Thermo Scientific Apreo S Hvac, Waltham, MA, USA). A solid ultraviolet spectrophotometer (UV-Vis, UV-3600, Persee, Beijing, China) was used to determine the UV-visible diffuse reflection spectrum of the samples. A specific surface area analyzer (BET, NOVA TOUCH LX1, Anton Paar, Catlettsburg, KY, USA) was used to measure the specific surface area and pore size distribution.

### 3.4. Photocatalytic Test

#### 3.4.1. Photocatalytic Degradation of Dye

The experiments were carried out in a DY-B photoreactor (Shanghai Deyang Yibang Instrument Co., LTD, Shanghai, China). The photoreactor consisted of a quartz cold trap reactor, a stirring device, and a light source (500 W Xenon lamp). PVA, PVA/CMCh, and g-CP of different masses of g-C_3_N_4_ (1 g 1 wt% g-CP, 0.5 g 2 wt% g-CP, 0.33 g 3 wt% g-CP, 0.25 g 4 wt% g-CP, 0.20 g 5 wt% g-CP) were mixed with 50 mL of RhB (4 mg L^−1^). The suspension was stirred in the dark for 80 min in order to obtain absorption–desorption equilibrium. After the dark reaction, the Xenon lamp was turned on and 4 mL suspension was centrifuged at regular intervals. An ultraviolet–visible spectrophotometer was used to detect the absorbance of supernatant at 554 nm.

#### 3.4.2. Zone of Inhibition Test

The hydrogels were immersed in deionized water to remove uncross-linked components and sterilized under UV light for 30 min. The OD value of *E. coli* (or *S. aureus*) was measured using an enzyme-labeled instrument at a wavelength of 600 nm (0.1 OD = 1 × 10^8^ CFU mL^−1^). The 0.1 mL suspension of the bacterial solution (1 × 10^6^ CFU mL^−1^) was evenly spread in LB Petri dish. Then, PVA, CMCh/PVA, and g-CP hydrogels with a diameter of 1 cm were placed above the medium. The bacteriostatic properties of the hydrogels were observed after 24 h.

#### 3.4.3. Photocatalytic Antibacterial Experiment

Firstly, 1 g of g-CP materials with different mass fractions was weighed and sterilized under a UV lamp. Then, 50 mL of *E. coli* suspension (1 × 10^6^ CFU mL^−1^) was added. A Xenon lamp at 500 W was used to simulate sunlight. The dark reaction was carried out for 80 min. Then, 0.1 mL of the bacterial solution was evenly spread on LB medium every 30 min during the light reaction stage and incubated at 37 °C for 24 h. Finally, an automated colony counter was used to count the number of colonies. The steps of photocatalytic antimicrobial experiments of hydrogels against *S. aureus* were the same as above.

#### 3.4.4. Cell Counting Kit-8 (CCK-8) Test

Mouse fibroblasts (L-929) were derived from Wuhan Punosai Life Science and Technology Co., Ltd., Wuhan, China. They were cultured in 5% CO_2_, 37 °C incubators using a 90% DMEM high-glucose medium. First, 1 g of materials was soaked in 10 mL of medium for 24 h to obtain their extracts. The adherent cells were digested with 2–3 mL of 0.25% trypsin for 1–2 min. After termination of digestion, the cell suspension was counted and diluted (1 × 10^5^ mL^−1^), and the cells were inoculated into 96-well culture plates. The cells were cultured overnight, and the hydrogel extracts were added. After 24 h of incubation, 10% CCK-8 enhancement solution was added and OD values were measured five times at 450 nm.

#### 3.4.5. Live and Dead Cell Staining Experiment

Cell viabilities were evaluated by using the Hoechst33342/Propidium lodide (PI) apoptosis detection kit. First, L-929 cells (1 × 10^5^ mL^−1^) were inoculated in 6-well plates and cultured overnight. The hydrogel extracts and cells were co-cultured for 24 h and washed twice using PBS buffer. Then, Hoechst33342 (10 mg L^−1^) solution was added and the cells were incubated in an incubator for 10 min. After the cells were washed twice with PBS, PI solution (10 mg L^−1^) was added and co-cultured for 20 min. After washing with PBS, blue and red fluorescence were detected at 400–500 nm and >630 nm, respectively.

## 4. Conclusions

To sum up, a multifunctional hydrogel was successfully developed using the electron beam radiation method. The structures and morphologies of g-CP were confirmed using FTIR, XRD, XPS, SEM, UV-vis DRS, and BET. Adsorption–degradation experiments showed that 1 wt% g-CP degraded RhB up to 65.92% in 60 min. The zone of inhibition and photocatalytic antimicrobial experiments proved that g-CP had a good dark–light dual-mode antibacterial effect on *E. coli* and *S. aureus*. At the same time, the cytotoxicity test proved that 2 wt% g-CP had good biocompatibility. More remarkably, 2 wt% g-CP had good mechanical properties (tensile and compressive moduli of 0.093 MPa and 1.61 MPa, respectively) and deformation recovery properties, which expands its applications in catalysis, engineering, and biomedical fields.

## Figures and Tables

**Table 1:** BET data of PVA, CMCh/PVA, and 2 wt% g-CP hydrogels.

Samples	Surface Area (m^2^ g^−1^)	Pore Volume (cc g^−1^)	Pore Diameter Dv (d) (nm)
PVA	0.132494	0.000239772	5.9785
CMCh/PVA	0.208232	0.000320396	3.85686
g-CP	4.3618	0.0541498	3.10875

**Table 2:** Kinetic parameters of RhB adsorption by PVA, CMCh/PVA, and g-CP hydrogels.

Absorbent	T (K)	q_e_, Exp (mg g^−1^)	Pseudo-First-Order			Pseudo-Second-Order		
			q_e_, Cal (mg g^−1^)	k_1_ (min^−1^)	R^2^	q_e_, Cal (mg g^−1^)	k_2_ (g mg^−1^ min^−1^)	R^2^
PVA	298	8.23	8.97	0.0653	0.9847	8.54	0.0128	0.9944
CMCh/PVA	298	10.9	10.02	0.0383	0.9853	11.12	0.0094	0.9961
g-CP	298	11.69	10.79	0.0342	0.995	12.15	0.0072	0.995

## Data Availability

Data are contained within the article.
